# Birth Rate Increases following Improved Rural Water Supply

**DOI:** 10.1371/journal.pmed.0030133

**Published:** 2006-02-14

Development efforts in rural Africa over the last few decades have achieved improvements in living conditions and in health. It has been argued that when such changes occur, there will be a subsequent reduction in birth rates, and experience in other parts of the developing world has tended to bear out this prediction. However, birth rates in rural Africa remain high and the population continues to grow rapidly. The situation in Ethiopia provides an illustration; spiraling population growth and slow economic growth are widely considered to be the main factors that have fuelled this country's repeated humanitarian crises. 

On the basis of current trends, it is predicted that Africa's population will double in the next 50 years, but in many countries, the resources are currently not available to sustain such a level of growth, and increased human suffering may be the consequence. An important question, but one that is seldom discussed, is whether development programs in Africa fail to make sustainable improvements over the long term because they lead to unsustainable increases in population growth rate. This concern is addressed, however, in a paper by Gibson and Mace, who studied a rural development program that was intended to improve the lives of Ethiopian women. The researchers measured its impact on the health of women and children in eight villages included within the program, and also on the birth rate. 

The study involved a rural area where some villages had benefited from the provision of a tapped water supply. Previously, women had to walk long distances (up to 30 km) to fetch their families' water in clay pots. The development program reduced the time they spent carrying water each day from about three hours to about 15 minutes. The researchers collected information over a four-year period, including for both villages where tapped water had been introduced and for others where it had not. In total, nearly 2,000 households were included. The nutritional status of the women and children (in terms of body mass index) was also measured. The researchers found that the availability of tapped water improved the survival of young children, although their nutritional status actually declined, and the birth rate increased. All this caused greater scarcity of resources within households.

The incremental effects of small changes to energy balance caused by development can increase strain on the household by increasing birth rates. This finding highlights the importance of continuing to improve access to contraception, especially in rural areas. 

